# Safety and effectiveness of tofacitinib in Korean adult patients with ulcerative colitis: post-marketing surveillance study

**DOI:** 10.1186/s12876-024-03336-2

**Published:** 2024-08-19

**Authors:** Hyuk Yoon, Byong Duk Ye, Sang-Bum Kang, Kang-Moon Lee, Chang Hwan Choi, Joo-young Jo, Juwon Woo, Jae Hee Cheon

**Affiliations:** 1 Seoul National University Bundang Hospital, Seongnam, Gyeonggi-do Korea; 2 University of Ulsan College of Medicine, Asan Medical Center, Seoul, Korea; 3 The Catholic University of Korea, Daejeon ST. Mary’s Hospital, Daejeon, Korea; 4 The Catholic University of Korea, ST. Vincent’s Hospital, Suwon, Gyeonggi-do Korea; 5 Chung-Ang University College of Medicine, Seoul, Korea; 6 Pfizer Pharmaceutical Korea Ltd, Seoul, Korea; 7 Department of Internal Medicine, College of Medicine, Yonsei University, 50-1, Yonsei-ro, Seodaemun-gu, Seoul, Republic of Korea

**Keywords:** Tofacitinib, Ulcerative colitis, Post-marketing surveillance, Prospective study, Safety, Effectiveness

## Abstract

**Background:**

Tofacitinib is an oral Janus kinase inhibitor for the treatment of ulcerative colitis (UC). We aimed to identify the safety and effectiveness of tofacitinib in patients with UC in routine clinical settings in Korea.

**Methods:**

This open-label, observational, prospective, post-marketing surveillance study was conducted at 22 hospitals in the Republic of Korea. Patients with moderate to severe active UC who received tofacitinib were included and followed up for up to 52 weeks. Tofacitinib was administered at a dosage of 10 mg twice daily for at least 8 weeks, followed by 5 or 10 mg twice daily at the investigator’s discretion based on clinical evaluation according to the approved Korean label. Safety including adverse events (AEs) and effectiveness including clinical remission, clinical response, and endoscopic mucosal healing were evaluated. Safety analysis set was defined as all patients registered for this study who received at least one dose of tofacitinib according to the approved Korean label and followed up for safety data. Effectiveness analysis set included patients in the safety analysis set who were evaluated for overall effectiveness assessment and excluded patients who had received tofacitinib less than 8 weeks.

**Results:**

A total of 110 patients were enrolled, of whom 106 patients were included in the safety population. The median duration of treatment was 370 days and the treatment duration ranged from 16 to 684 days for the safety population. AEs occurred in 42 patients (39.6%). Serious AEs (SAEs) occurred in 7 patients (6.6%) and of them, there were 2 cases of serious infections. These serious infections were reported as Adverse Event of Special Interest (AESI) in this study and no other AESI were reported. There were no cases of death during the study period. Clinical remission rates were 40.0%, 46.7%, 57.6%, and 55.1% at 8, 16, 24, and 52 weeks, and clinical response rates were 77.8%, 87.9%, 56.6%, and 81.4% at each visit, respectively. Endoscopic mucosal healing rates were 58.7% at 16 weeks and 46.2% at 52 weeks.

**Conclusion:**

Tofacitinib was effective in Korean patients with moderate to severe active UC and the safety findings were consistent with the known safety profile of tofacitinib.

**Summary:**

This study confirmed the safety and effectiveness of tofacitinib in Korean patients with moderate to severe active UC in routine clinical settings.

**Trial registration:**

This study is registered in the ClinicalTrials.gov under the identifier NCT04071405, registered on 28 August 2019.

## Background

Ulcerative Colitis (UC) is a chronic inflammatory disease of the colorectum and the extent of the disease is variable. It most commonly presents with rectal bleeding and diarrhea and is characterized by periodic relapse and remission of mucosal inflammation [1]. Treatment options for UC include corticosteroids, aminosalicylates, immunosuppressant such as azathioprine and 6-mercaptopurine, and biologics such as tumor necrosis factor (TNF) inhibitors, vedolizumab, and ustekinumab [2]. The introduction of TNF inhibitors has improved the clinical outcomes and quality of life in patients with UC by less hospitalizations or surgeries, and greater clinical remission and mucosal healing rates as well as symptomatic improvement [3–6]. However, the rate of primary or secondary failure to anti-TNF therapies still remains high in patients with UC. Up to 40% of patients who receive TNF inhibitor therapy fail to respond to induction dosing, and up to 46% of patients experience loss of response to TNF inhibitors [6–8].

Tofacitinib is an oral Janus kinase (JAK) inhibitor and inhibits JAK1, JAK2, JAK3 and, to a lesser extent, tyrosine kinase 2 (TYK2) [9]. In cellular settings, where JAKs signal in pairs, tofacitinib preferentially inhibits signaling by cytokine receptors associated with JAK3 and/or JAK1 with functional selectivity over receptors that signal via pairs of JAK2 [9]. JAK mediates signal transduction pathways for several cytokines such as pro-inflammatory cytokines involved in the pathogenesis of inflammatory diseases [10–13]. The efficacy and safety of tofacitinib in patients with moderate to severe UC have been evaluated in clinical trials [14–19]. In a phase 2 induction study, [14] two phase 3 induction studies (OCTAVE Induction 1 and 2), a phase 3 maintenance study (OCTAVE Sustain), [15] an open-label, extension study (OCTAVE Open) [16] and a phase 3b/4 study (RIVETING), [17] tofacitinib has been shown to be effective for both induction and maintenance of remission and mucosal healing. Furthermore, the effectiveness and safety of tofacitinib were confirmed in real-world studies in patients with moderate to severe active UC [18].

In controlled trials, rare side effects or adverse events in special situations and in patients with long-term comorbidity may not become apparent since patients who are ineligible for the controlled trials such as women of potential child-bearing, the elderly, and patients with comorbid conditions are excluded from the studies. Therefore, post-marketing surveillance (PMS) study is important to further characterize the safety profile of a product after launch under routine clinical practice [20, 21]. This PMS study is aimed to evaluate the safety and effectiveness of tofacitinib in patients with moderate to severe active UC during routine clinical practice in the Republic of Korea.

## Methods

### Study design and treatment

This open-label, observational, prospective, PMS study was conducted at 22 hospitals in the Republic of Korea to evaluate the safety and effectiveness of tofacitinib in patients with UC. In total, 110 patients participated in the study from 20 September 2018 to 19 September 2022. Adult patients who received at least one dose of tofacitinib for the treatment of moderate to severe active UC who have had an inadequate response or intolerance to the basic treatments or biological agents were enrolled and followed up for up to 52 weeks after the first treatment of tofacitinib. Patients with a history of hypersensitivity to any ingredients of this product (Tofacitinib Tablets 5 mg, 10 mg), those with current serious or active infections including localized infection active tuberculosis, those with severe hepatic function disorder, an absolute neutrophil count (ANC) (< 1,000 cells/mm^3^), a lymphocyte count (< 500 cells/mm^3^), a hemoglobin level (< 9 g/dL) or hereditary problems including galactose intolerance, Lapp lactase deficiency or glucose-galactose malabsorption, and pregnant or possibly pregnant women were excluded. Tofacitinib was administered at a dosage of 10 mg twice daily for at least 8 weeks, followed by 5 or 10 mg twice daily at the investigator’s discretion based on clinical evaluation according to the approved Korean label. The variables for patient demographics and baseline characteristics were age, sex, height, weight, disease duration, disease severity, latent tuberculosis, herpes zoster (HZ) vaccination, smoking status, previous UC treatment, and concomitant medication. Disease severity was defined as severe (Mayo Score 11–12, Partial Mayo Score 7–9), moderate (Mayo Score 6–10, Partial Mayo Score 5–6), mild (Mayo Score 3–5, Partial Mayo Score 2–4) and remission (Mayo Score 0–2, Partial Mayo Score 0–1). Demographics and baseline characteristics were investigated through medical records of each patient or by asking patients.

### Assessments

#### Safety

Safety was assessed according to adverse events (AEs) reported throughout the study period from all patients who received at least one dose of tofacitinib. The severity of AEs was categorized as mild (not causing any significant problem to the patient. Administration of medicinal product continues without dose adjustment), moderate (causes a problem that does not interfere significantly with usual activities or the clinical status. Dose of the medicinal product is adjusted, or other therapies are added due to the AE) and severe (causes a problem that interferes significantly with usual activities or the clinical status. The medicinal product is stopped due to the AE). Serious AEs (SAEs) were defined as life-threatening AEs or AEs resulting in death, inpatient hospitalization or prolongation of hospitalization, persistent or significant disability/in capacity, or congenital anomaly/birth defect. The AEs of special interest comprised serious infection, tuberculosis, malignancy, or lymphocyte proliferative disorders. AEs were coded using the Medical Dictionary for Regulatory Activities (MedDRA) version 25.0.

#### Effectiveness

Effectiveness was assessed at baseline, 8, 16, 24, and 52 weeks by the proportion of patients achieving clinical remission, clinical response, endoscopic mucosal healing, steroid-free clinical remission and steroid-free clinical response as it was observed. Clinical remission was defined as Mayo score of ≤ 2 (in case of partial Mayo score, the score of ≤ 1) and no subscores > 1 and rectal bleeding score of 0 [15]. Clinical response was defined as a decrease in Mayo score of ≥ 3 points and 30% from the baseline and a decrease of ≥ 1 in rectal bleeding score or rectal bleeding score of 0 or 1 (in case of partial Mayo score, a decrease of partial Mayo score of ≥ 2 and 30% from the baseline and decrease of ≥ 1 in rectal bleeding score or rectal bleeding score of 0 or 1) [15]. Endoscopic mucosal healing was defined as a Mayo endoscopic score (MES) of 0 or 1 [15]. Steroid-free clinical remission/response were defined as the clinical remission/response status without the use of systemic corticosteroids at the time of evaluation.

### Statistical analyses

The safety population included all patients who received at least one dose of tofacitinib and followed up for safety information. The effectiveness population included all patients in the safety population who received tofacitinib for at least 8 weeks and had an overall effectiveness assessment by the investigator. Continuous variables were summarized by descriptive statistics including n, mean, Standard deviation (SD), median, minimum, maximum, and categorial variables were presented in frequency and percentage. Changes in MES from baseline to 52 weeks were tested by using Wilcoxon signed rank test. All statistical analyses were conducted using SAS software, version 9.4 (SAS institute, Cary, NC, USA).

## Results

### Patient disposition and demographics

**Fig. 1:**
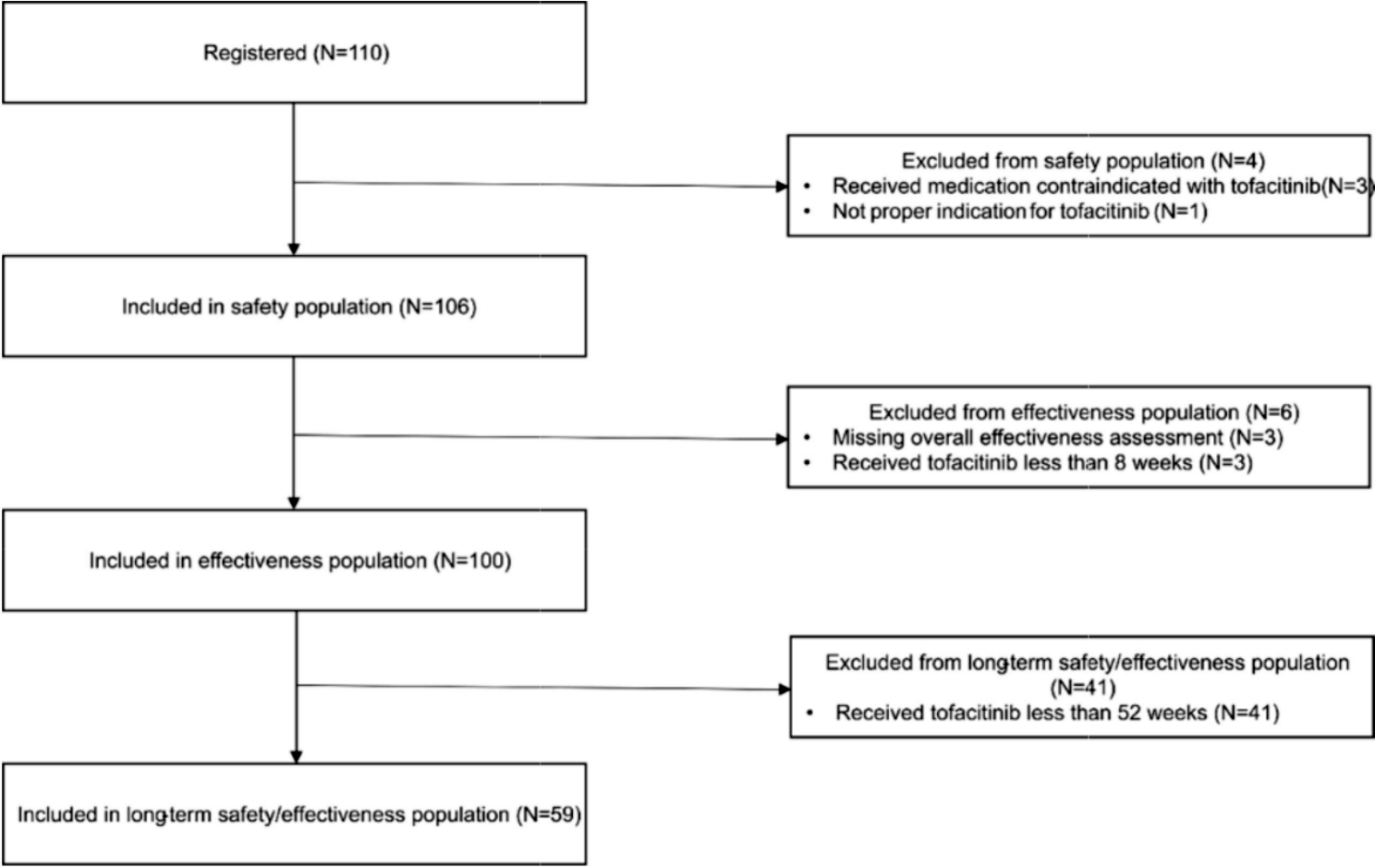
Patient disposition of the study

The patient disposition is presented in Fig. 1. Out of 110 patients, 106 patients were included in the safety population. Among the 106 patients, a total of 100 patients were included in the effectiveness population. The median age of the patients was 39.0 years (Interquartile range (IQR) 20) and 75 patients (70.8%) were male. The median disease duration was 5 years (range, 0.3–27.0), and 15 patients (14.2%) and 91 patients (85.8%) were severe and moderate in disease severity, respectively.

**Fig. 2:**
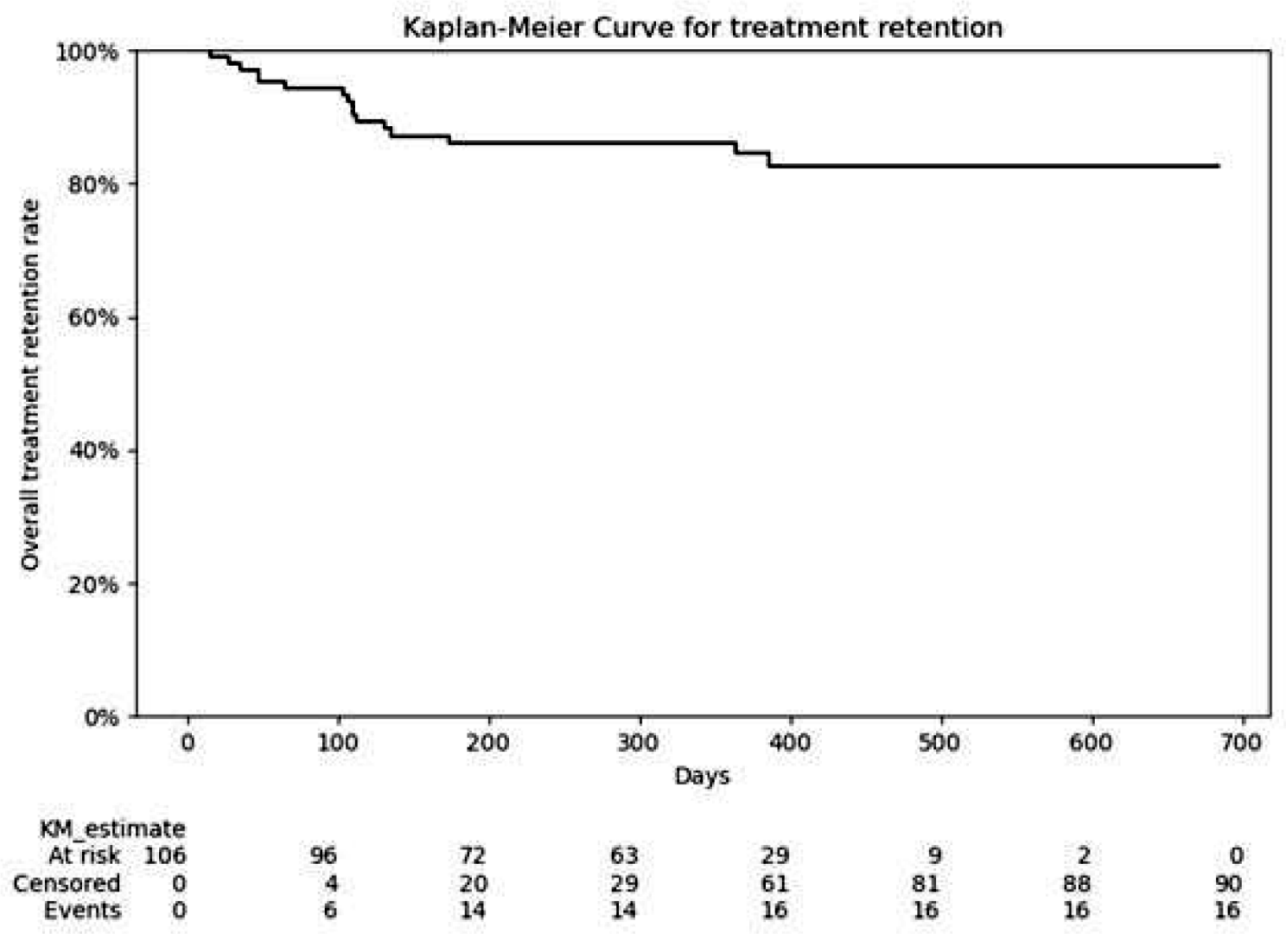
Treatment retention rate for all patients in the study

10 patients (9.4%) had a history of latent tuberculosis at baseline. For patients with latent tuberculosis, the treatment for latent tuberculosis were preceded before administration of tofacitinib. All the 106 patients (100.0%) had both previous UC treatments and concomitant medications; 44 patients (41.5%), 93 patients (87.7%), 39 patients (36.8%), and 52 patients (49.1%) had previously been treated with systemic corticosteroids, 5-aminosalicylic acids, Thiopurines, and biologic agents, respectively. Concomitant systemic corticosteroids were prescribed to 35 patients (33.0%) (Table 1). The median duration of treatment was 370 days and the treatment duration ranged from 16 to 684 days. The overall treatment retention rate was 84.9% (90/106) during the study period (Fig. 2). Among the 101 patients undergoing maintenance therapy of tofacitinib, patients undergoing maintenance therapy by 5 mg bid of tofacitinib were 44 patients (43.6%), while those undergoing maintenance therapy by 10 mg bid of tofacitinib were 56 patients (55.4%). The patient undergoing maintenance therapy by 20 mg bid of tofacitinib was one patient (1.0%).

**Table 1:** Patient demographics & baseline characteristics

Characteristic		*n* = 106
Age, median (IQR), years (range)		39.0 (20.0) (20.0 ~ 73.0)
Sex, n (%)	Male	75 (70.8)
	Female	31 (29.2)
Height,^†^ mean (SD), cm		170.14 (9.7)
Weight,^†^ mean (SD), kg		65.46 (14.9)
Disease duration,^§^ median (range), years		5 (0.3 ~ 27.0)
Disease severity,^¶^ n (%)	Severe	15 (14.2)
	Moderate	91 (85.8)
Latent tuberculosis, n(%)	Yes	10 (9.4)
	No	95 (89.6)
	Unknown	1 (0.9)
Herpes zoster vaccination, n(%)	Yes	5 (4.7)
	No	38 (35.8)
	Unknown	63 (59.4)
Smoking, n(%)	Ex-smoker	25 (23.6)
	Current smoker	13 (12.3)
	Non-smoker	55 (51.9)
	Unknown	13 (12.3)
Previous UC treatment,^‡^ n(%)		106 (100.0)
	Systemic corticosteroids	44 (41.5)
	5-aminosalicylic acids	93 (87.7)
	Thiopurines	39 (36.8)
	Biologics	52 (49.1)
	TNF inhibitors^††^	32 (30.2)
	Vedolizumab	21 (19.8)
	None (biologics-naïve)	54 (50.9)
Concomitant medication,^‡^ n(%)		106 (100.0)
	Systemic corticosteroids	35 (33.0)

^†^Height unknown: 12 subjects, Weight unknown: 11 subjects, Disease duration unknown: 2 subjects

^‡^Overlapped

^§^The duration from when ulcerative colitis was first diagnosed until the day that first dose of tofacitinib is taken

^¶^Severe: Mayo Score 11–12, Partial Mayo Score 7–9, Moderate: Mayo Score 6–10, Partial Mayo Score 5–6, Mild: Mayo Score 3–5, Partial Mayo Score 2–4, Remission: Mayo Score 0–2, Partial Mayo Score 0–1

^††^TNF inhibitors: Infliximab, Adalimumab or Golimumab

*Abbreviations* IQR; Interquartile range; n, number of patients; SD, standard deviation; TNF, tumor necrosis factor; UC, ulcerative colitis

### Safety

Of the 106 patients, 42 patients (39.6%) experienced a total of 85 AEs. The most common AEs were UC aggravation, which occurred in 7 patients (6.6%, 8 events) and anemia in 7 patients (6.6%, 7 events). Eight SAEs occurred in 7 patients (6.6%), who all required inpatient hospitalization or prolongation of hospitalization. Of these, eight events were assessed as unlikely to be related to tofacitinib treatment by investigators with the exception of one event (drug ineffective). There were no deaths (Table 2). Out of 85 AEs, the majority of AEs were mild (64 events [75.3%]) or moderate (17 events [20.0%]) in severity, and severe (4 events [4.7%]) AEs were UC aggravation and abdominal pain.

**Table 2:** AEs and SAEs during tofacitinib treatment

Adverse Event	AEs			SAEs		
	Number of patients, *n* (%)	Number of AEs	Incidence rates per 100PY^†^	Number of patients, *n* (%)	Number of SAEs	Incidence rates per 100PY^†^
Any event^‡^	42 (39.6)	85	47.2	7 (6.6)	8	7.9
Ulcerative Colitis^§^	7 (6.6)	8	7.9	2(1.9)	3	2.3
Anemia	7 (6.6)	7	7.9	0 (0.0)	0	NA
Dyslipidemia	4 (3.8)	4	4.5	0 (0.0)	0	NA
Myalgia	3 (2.8)	4	3.4	0 (0.0)	0	NA
Headache	3 (2.8)	3	3.4	0 (0.0)	0	NA
Pyrexia	3 (2.8)	3	3.4	0 (0.0)	0	NA
Cytomegalovirus colitis	2 (1.9)	3	2.3	1 (0.9)	1	1.1
Abdominal pain	2 (1.9)	2	2.3	1 (0.9)	1	1.1
Aspartate aminotransferase increased	2 (1.9)	2	2.3	0 (0.0)	0	NA
Blood cholesterol increased	2 (1.9)	2	2.3	0 (0.0)	0	NA
COVID-19	2 (1.9)	2	2.3	1 (0.9)	1	1.1
Drug ineffective	2 (1.9)	2	2.3	1 (0.9)	1	1.1
Rash	2 (1.9)	2	2.3	0 (0.0)	0	NA
Anal abscess	1 (0.9)	2	1.1	1 (0.9)	1	1.1

MedDRA 25.0 (MedDRA-K 25.0)

^†^Total exposure was 90.3 patient-years

^‡^Preferred term with < 1% of AEs which were not reported as SAEs are not shown

^§^Lowest level term of ulcerative colitis was colitis ulcerative aggravated or UC aggravated

*Abbreviations* AE, adverse event; COVID-19, coronavirus disease 2019; PY, patient-years of exposure; SAE, serious adverse event; NA, not applicable

In AEs of special interest (serious infection, tuberculosis, malignancy, lymphocyte proliferative disorders), serious infections were observed in 2 patients (1.9%, 2 events), which were anal abscess and cytomegalovirus colitis, respectively. No cases of tuberculosis, malignancy, or lymphocyte proliferative disorders were reported (Table 3). In addition, none of HZ, major adverse cardiovascular event (MACE), and venous thromboembolism (VTE) were reported. There was no statistically significant difference in AEs rates by age group (*p* = 0.1456).

**Table 3:** AEs of special interest^†^

Adverse Event	Adverse events of special interest			
	Number of patients, *n* (%)	Number of AEs	Incidence rates per 100PY^‡^	
Serious infections	2 (1.9)	2	2.3	
Anal abscess	1 (0.9)	1	1.1	
Cytomegalovirus colitis	1 (0.9)	1	1.1	
Tuberculosis	0 (0.0)	0	NA	
Malignancy	0 (0.0)	0	NA	
Lymphocyte proliferative disorders	0 (0.0)	0	NA	

MedDRA 25.0 (MedDRA-K 25.0)

^†^AEs of special interest is defined as events related to important identified risks or important potential risks, including serious infection, tuberculosis, malignancy and lymphocyte proliferative disorders

^‡^Total exposure was 90.3 patient-years

*Abbreviations* AE, adverse event; PY, patient-years of exposure; NA, not applicable

### Effectiveness

**Fig. 3:**
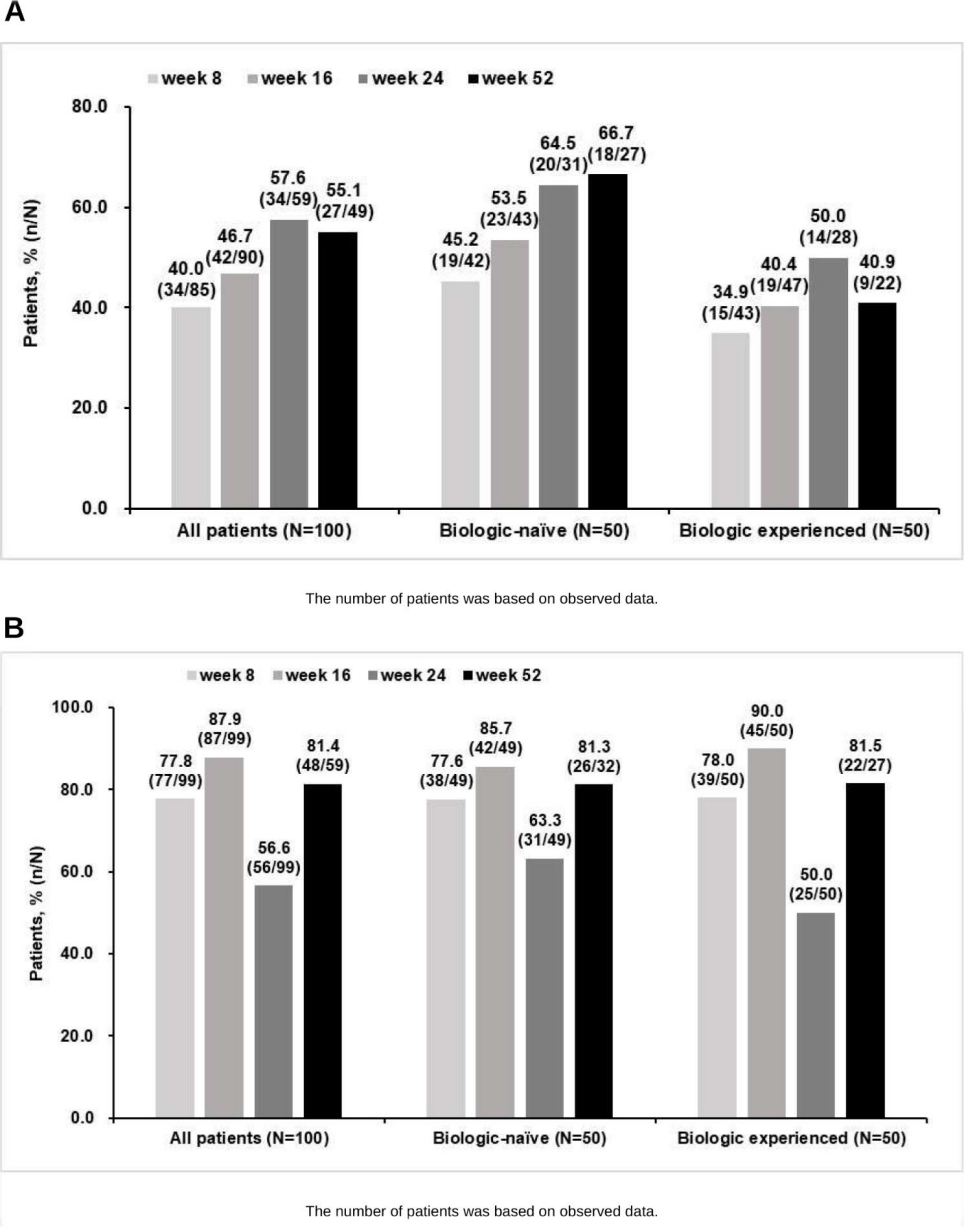
Proportion of patients achieving (**A**) clinical remission; and (**B**) clinical response at 8, 16, 24 and 52 weeks

The number of patients who have evaluated at each time point for each effectiveness endpoint is used as a denominator calculating proportions of patients achieving clinical remission, clinical response. Since this study was a non-interventional study conducted under routine clinical practice and there were no fixed visits, the number of patients evaluated for each effectiveness endpoint (clinical remission, clinical response, endoscopic mucosal healing rate) and each time point was different. Therefore, differences occurred in the denominator at each time point within each effectiveness endpoint. In the effectiveness population, according to observed data, proportions of patients who achieved clinical remission were 40.0% (34/85 patients), 46.7% (42/90 patients), 57.6% (34/59 patients), and 55.1% (27/49 patients) at 8, 16, 24, and 52 weeks, respectively (Fig. 3A) and those who achieved clinical response were 77.8% (77/99 patients), 87.9% (87/99 patients), 56.6% (56/99 patients), and 81.4% (48/59 patients) at 8, 16, 24 and 52 weeks, respectively (Fig. 3B).

**Fig. 4:**
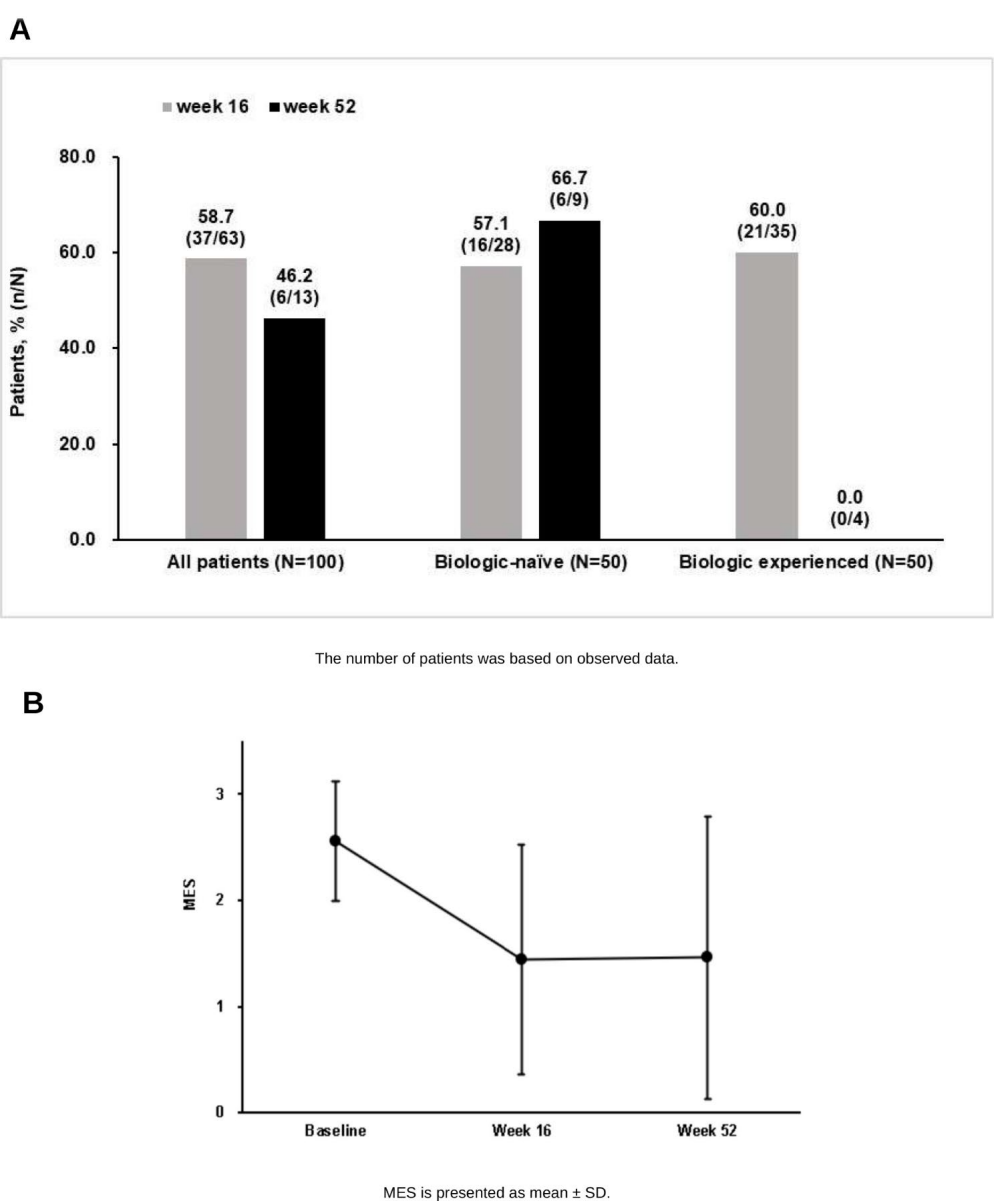
Proportion of patients achieving (**A**) endoscopic mucosal healing at 16 and 52 weeks; and (**B**) the changes in Mayo endoscopic score (MES) to 52 weeks

Proportions of patients who achieved endoscopic mucosal healing were 58.7% (37/63 patients) and 46.2% (6/13 patients) at 16 weeks and 52 weeks, respectively (Fig. 4A). When we compared measured values from the last value to those from baseline by each patient, MES was decreased from baseline to 16 weeks (*p* < 0.001) and maintained until 52 weeks with a significant difference from baseline (*p* < 0.001) (Fig. 4B). The proportions of patients who achieved clinical remission at 8, 16, 24, and 52 weeks and endoscopic mucosal healing at 52 weeks were numerically higher in biologic-naïve patients than biologic-experienced patients (Figs. 3A and 4A). Proportions of clinical response were similar between biologic-naïve patients and biologic-experienced patients at all time points (Fig. 3B).

Steroid-free clinical remission and steroid-free clinical response is presented in Table 4. At week 8, steroid-free clinical remissions of 71 patients were evaluated and 30 patients (42.3%) achieved steroid-free clinical remission. At week 16, steroid-free clinical remissions of 77 patients were evaluated, and 38 patients (49.4%) achieved steroid-free clinical remission. At week 24, steroid-free clinical remissions of 56 patients were evaluated and 32 patients (57.1%) achieved steroid-free clinical remission. At week 52, steroid-free clinical remissions of 46 patients were evaluated and 26 patients (56.5%) achieved steroid-free clinical remission.

**Table 4:** Steroid-free clinical remission, clinical response

		Number of subjects (%)							
		Week 8 (± 15 days) *n* (%)		Week 16 (± 15 days) *n* (%)		Week 24 (± 15 days) *n* (%)		Week 52 (± 15 days) *n* (%)	
Clinical Remission	Achieved	30	(42.3)	38	(49.4)	32	(57.1)	26	(56.5)
	Not achieved	41	(57.7)	39	(50.6)	24	(42.9)	20	(43.5)
	Total	71	(100.0)	77	(100.0)	56	(100.0)	46	(100.0)
Clinical Response	Achieved	66	(80.5)	74	(86.0)	53	(57.0)	45	(80.4)
	Not achieved	16	(19.5)	12	(14.0)	40	(43.0)	11	(19.6)
	Total	82	(100.0)	86	(100.0)	93	(100.0)	56	(100.0)

n: Number of subjects

Also, steroid-free clinical responses of 82 patients were evaluated at week 8 and 66 patients (80.5%) achieved steroid-free clinical response. At week 16, steroid-free clinical responses of 86 patients were evaluated and 74 patients (86.0%) achieved steroid-free clinical response. At week 24, steroid-free clinical responses of 93 patients were evaluated and 53 patients (57.0%) achieved steroid-free clinical response. At week 52, steroid-free clinical responses of 56 patients were evaluated and 45 patients (80.4%) achieved steroid-free clinical response (Table 4).

## Discussion

This PMS study aimed to assess the safety and effectiveness of tofacitinib in patients with moderate to severe active UC during routine clinical practice in the Republic of Korea. We evaluated AEs and effectiveness including clinical remission, clinical response, and endoscopic mucosal healing for 52 weeks after the first dose of tofacitinib. Overall, tofacitinib was well tolerated and the safety results were consistent with the known safety profile of tofacitinib. Approximately, 40% of patients experienced AEs, which were mostly mild or moderate in severity. The most common AE was UC aggravation as reported in previous randomized controlled studies of tofacitinib and a real-world study [15–18, 22]. The risk of serious infections including HZ has been reported in patients with tofacitinib [23–28]. In this PMS study, there were two serious infections including anal abscess and cytomegalovirus colitis but there was no case of HZ. At baseline, 4.7% (5/106 patients) of the safety population had a history of HZ vaccination, 35.8% (38/106 patients) had no history of HZ vaccination, and 59.4% (63/106 patients) were unknown. Information on HZ vaccination during tofacitinib treatment was not collected. Since the proportion of patients whose HZ vaccination status was unknown was high, it could not be concluded that HZ vaccination is the main reason for no case of HZ in this study. Recently, the incidence rate of HZ was reported as 3.19/100 patient-years in a retrospective real-world study of tofacitinib in a Korean cohort with UC [29]. In tofacitinib UC clinical program, the incidence rate of HZ was 3.38/100 patient-years [30]. The reasons why this study showed the different trend of occurrence for HZ cases may include differences in patient characteristics, especially those with identified risk factors for HZ such as older age and prior TNF inhibitor failure [27, 30]. Although no case of HZ was reported in this study, this does not suggest that HZ should be excluded from the risk of tofacitinib since information on HZ vaccination and the sample size were limited. In addition, tofacitinib has been associated with the risk of tuberculosis, malignancy, and lymphocyte proliferative disorders in rheumatoid arthritis or in UC infrequently, [25, 31–35] but none of those cases were reported in this study.

A Korean Association for the Study of Intestinal Diseases (KASID) multicenter cohort study was the retrospective observational study and analyzed the data of 148 patients with UC who received tofacitinib treatment at 12 hospitals in Korea between January 2018 and November 2020 [29]. It showed proportions of patients who achieved clinical remission 60.6% (86/142 patients), 54.9% (78/142 patients) and 52.8% (75/142 patients) at 16, 24, 52 weeks, respectively and those who achieved clinical response were 71.8% (102/142 patients), 67.6% (96/142 patients), 59.9% (85/142 patients) at 16, 24, 52 weeks, respectively [29]. The results of effectiveness analysis had differences by each time points of this study but most of them showed a similar tendency. In safety analysis, AEs were reported to be 12.8% (19/148 patients), which was lower than that of this study. However, SAEs and cytomegalovirus colitis were reported to be 8.1% (12/148 patients), 4.1% (4/148 patients) respectively, which were higher than those of this study. In the KASID study, considering of retrospective study design we assessed that SAE interested by clinicians had been collected appropriately but there was a possibility that general AE could had been collected relatively less. Therefore, we assessed that safety results of tofacitinib in this study was more reliable considering of the prospective study design which could collect and monitor AEs closely during the study period.

In the OCTAVE Induction trial, clinical remission and clinical response rates were 16.6-18.5% and 55.0-59.9% at 8 weeks, respectively. In the OCTAVE Sustain trial, the clinical remission rate was 34.3-40.6%, and the clinical response rate was 51.5-61.9% at 52 weeks [15]. In the effectiveness analyses of this study, clinical remission rates were 40.0%, 46.7%, 57.6%, and 55.1%, and clinical response rates were 77.8%, 87.9%, 56.6%, and 81.4% at 8, 16, 24 and 52 weeks, respectively. These results showed maintained clinical remission and response to 52 weeks after starting tofacitinib treatment in patients who remained in the study, and comparable effectiveness to those reported in other real-world studies. Taxonera C, et al. [18] reported the meta-analysis of real-world studies of tofacitinib for investigating safety and effectiveness, which included seventeen studies with a total of 1,162 patients with moderate to severe active UC; Clinical remission was achieved in 34.7% of patients at 8 weeks and in 47% at 12–16 weeks. At 6 and 12 months, 38.3% and 41.4% of patients were in clinical remission, respectively. Clinical response was achieved in 62.1% of patients at 8 weeks and in 64.2% at 12–16 weeks. At 6 and 12 months, 50.8% and 41.8% of patients had sustained response, respectively [18]. Our study also showed similar endoscopic outcomes to those of previous studies, that is, endoscopic mucosal healing rates were 58.7% and 46.2% at 16 and 52 weeks, respectively. In OCTAVE trial, endoscopic mucosal healing was achieved in 28.4-31.3% of patients at 8 weeks and in 37.4-45.7% of patients at 52 weeks [15]. In real-world studies, 50.0-64.9% of patients achieved endoscopic mucosal healing at 12–16 weeks [36, 37]. In the effectiveness analysis of this study according to prior biologic exposure, the effectiveness of tofacitinib was confirmed in both biologic-naïve and experienced patients. Clinical response was similar between the two cohorts at 8, 16, 24, and 52 weeks. In biologic-naïve patients, clinical remission and mucosal healing showed numerically higher trends during the study period than those in biologic-experienced patients; however, cautious interpretation with this finding may be needed to conclude the better effectiveness of tofacitinib in biologic-naïve patients than in experienced patients.

Tofacitinib was approved as a treatment for UC in September 2018 in Korea, and it included all biologic-naïve and experienced patients. Since ORAL surveillance study [33], the use of JAK inhibitor was restricted to biologic-inadequate responder in high-risk patients (patients 65 years and older, at high risk for cardiovascular disease, at risk for malignancy), and it is still available in biologic-naïve in other patients. In this PMS study, the recruitment of patients was conducted from September 2018 to September 2022, and the label change in Korea was announced in June 2022. Therefore, all biologic-naïve and experienced patients were recruited in this PMS study.

In OCTAVE trial, clinical remission rates at 8 weeks were almost double in TNF inhibitor-naïve patients compared to TNF inhibitor-experienced patients and endoscopic mucosal healing rates at 8 weeks were also higher in TNF inhibitor-naïve patients than in TNF inhibitor-experienced patients, but the differences of rates of both clinical remission and endoscopic mucosal healing between placebo and tofacitinib treatment were similar in TNF inhibitor-naïve and experienced patients [15]. A real-world study for the effectiveness of tofacitinib also showed similar corticosteroid-free clinical remission between biologic-naïve and experienced patients [38]. Thus, further research is needed to establish evidence for comparing the effectiveness of tofacitinib between biologic-naïve and experienced patients given the differences between studies such as design and study population.

This study evaluated the safety and effectiveness of tofacitinib in patients with moderate to severe active UC; however, the findings of this study should be interpreted with care considering the several potential limitations of this study; First, the size of the study population was relatively small; the number of patients in safety analysis and effectiveness analysis was 106 and 100, respectively. Besides, only 13 patients were assessed at week 52 on endoscopic mucosal healing; this also meant a lack of objective biochemical data. Second, there was no comparative arm or placebo in this observational study; therefore, the effectiveness could be overestimated. Conversely, this study has the strength that the observational study is more likely to reflect clinical practice compared to the randomized controlled trials in terms of the heterogeneous populations and medications which patients have received. In addition, AEs were closely monitored and collected during the study period in terms of the nature of the prospective study. As a result, there were no AEs such as HZ, MACE and VTE which have been reported as increased risks in patients with tofacitinib treatment or in patients with inflammatory bowel disease (IBD) [39]. Therefore, the results of this study provide evidence of safety and effectiveness of tofacitinib in patients with moderate to severe active UC.

## Conclusions

The results of the study showed an acceptable safety consistent with known safety profile of tofacitinib and effectiveness with for Korean patients with moderate to severe active UC in routine clinical practice.

## Data Availability

The data that support the findings of this study are available from Pfizer Pharmaceutical Korea Ltd, but restrictions apply to the availability of these data, which were used under license for the current study, and so are not publicly available. Data are however available from the authors upon reasonable request and with permission of Pfizer Pharmaceutical Korea Ltd.

## Abbreviations

AE: Adverse Event
AESI: Adverse Event of Special Interest
ANC: Absolute Neutrophil Count
GPP: Good Pharmacoepidemiology Practice
HZ: Herpes Zoster
IBD: Inflammatory Bowel Disease
IRB: Institutional Review Board
IQR: Interquartile range
ISPE: International Society for Pharmacoepidemiology
JAK: Janus kinase
KASID: Korean Association for the Study of Intestinal Diseases
MACE: Major Adverse Cardiovascular Event
MedDRA: Medical Dictionary for Regulatory Activities
MES: Mayo Endoscopic Score
MFDS: Ministry of Food and Drug Safety
PhRMA: Pharmaceutical Research and Manufacturers Association
PMS: Post-marketing Surveillance
SAE: Serious Adverse Event
SD: Standard deviation
TNF: Tumor Necrosis Factor
TYK: Tyrosine kinase
UC: Ulcerative Colitis
VTE: Venous thromboembolism
